# Anoikis in Gastric Cancer: Mechanistic Insights Into Metastatic Survival and Therapeutic Targeting

**DOI:** 10.1002/jbt.71167

**Published:** 2026-11-15

**Authors:** Jian Zhong, Xing Qi, Honglei Wu, Zheng Lei

**Affiliations:** ^1^ General Medicine Department Ziyang Central Hospital Ziyang China; ^2^ Department of Gastroenterology College of Clinical Medicine of Henan University of Science and Technology luoyang China; ^3^ Intensive Care Unit Ziyang Central Hospital Ziyang China

**Keywords:** anoikis resistance, gastric cancer, integrin/FAK signaling, peritoneal metastasis, PI3K‐AKT, therapeutic targeting

## Abstract

Gastric cancer (GC) remains a major cause of cancer‐related mortality worldwide, largely because many patients present with advanced or metastatic disease. Anoikis, a specialized form of detachment‐induced apoptosis, is a critical safeguard that eliminates epithelial cells after loss of appropriate extracellular matrix (ECM) and cell–cell adhesion; therefore, its disruption allows malignant cells to survive outside their native niche and acquire metastatic competence. In GC, anoikis resistance is central to tumor progression and peritoneal dissemination, enabling detached tumor cells to survive anchorage loss, mechanical stress, metabolic adaptation, and immune pressure before colonization. This review synthesizes current evidence on how anoikis is initiated, executed, and evaded in GC. Mechanistically, anoikis initiation begins with disruption of integrin–ECM and cadherin‐mediated adhesion, followed by focal adhesion remodeling, cytoskeletal reorganization, and suppression or rewiring of adhesion‐dependent survival signaling. Anoikis execution converges on mitochondrial and death‐receptor apoptotic pathways, including Bcl‐2 family regulation, caspase‐8/caspase‐9 activation, and executioner caspase cleavage. In contrast, anoikis evasion is driven by interconnected survival hubs, including integrin/FAK/Src, PI3K‐AKT, MEK‐ERK, NF‐κB, Wnt/β‐catenin, YAP/TEAD, BDNF/TrkB, and protective autophagy. GC‐specific mediators such as TCF7L2/PLAUR, MYH9/CTNNB1, ITGBL1/AKT/FBLN2, CAV1/epidermal growth factor receptor (EGFR)/ITGB1, GRP94/YAP–TEAD1, GDF15/autophagy, and TRIM69/PRKCD/BDNF further illustrate how detached GC cells acquire metastatic survival capacity. Translationally, these pathways provide a rationale for biomarker‐guided therapeutic strategies aimed at restoring anoikis sensitivity or disabling compensatory survival programs. Targeting anoikis resistance may therefore complement existing chemotherapy, targeted therapy, and immunotherapy approaches, particularly in GC subtypes characterized by peritoneal dissemination, EMT, YAP/TEAD activation, or integrin/PI3K‐AKT pathway dependence.

## Graphical abstract

The correlation between anoikis resistance and the spread of cancer cells. “Anoikis” refers to a form of cell death that transpires when cells disengage from their neighboring stromal cells. The ability to resist anoikis is a defining characteristic of cancer cell migration, as these cells must demonstrate resilience after becoming detached from their supportive cellular environment.

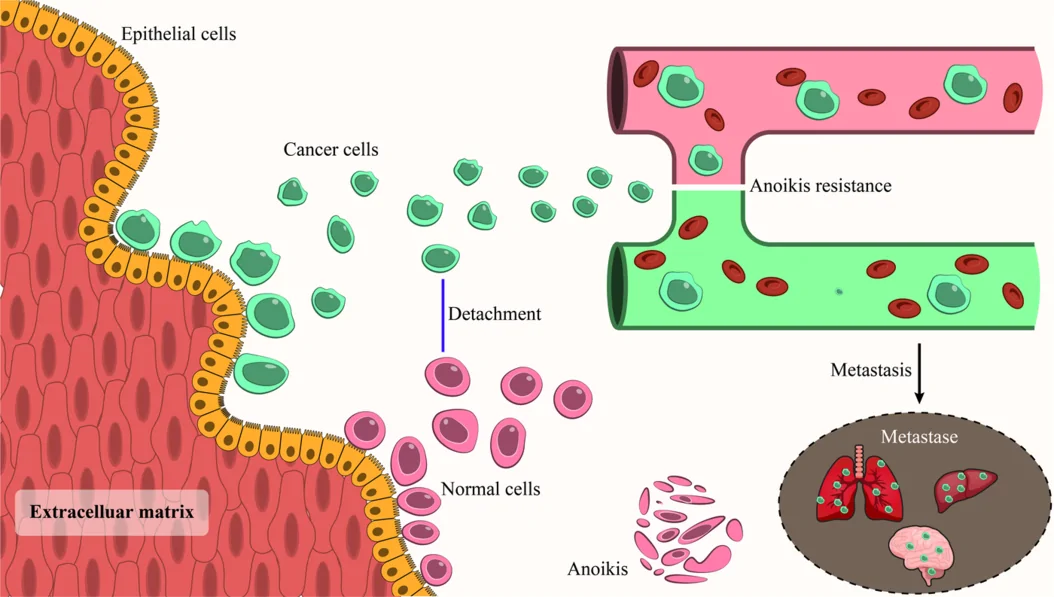

## 1 Introduction

Gastric cancer (GC) remains a major global health burden, with more than 968,000 new cases and 659,000 deaths estimated worldwide in 2022, ranking among the leading causes of cancer‐related mortality [1, 2]. The poor clinical outcome of GC is closely linked to advanced‐stage presentation and metastatic dissemination. Among metastatic routes, peritoneal dissemination is particularly challenging because it requires tumor cells to detach from the primary lesion, survive in the free peritoneal cavity or ascitic fluid, and subsequently adhere to and colonize the mesothelial surface [3, 4, 5]. These clinical and biological features provide a direct rationale for focusing on anoikis resistance, because only detached GC cells that overcome detachment‐induced apoptosis, termed anoikis in the context of adhesion loss, can persist during dissemination and establish metastatic lesions [6, 7, 8, 9].

Anoikis is a specialized form of programmed cell death triggered by the loss of appropriate cell–extracellular matrix (ECM) attachment or disruption of normal cell–cell adhesion [10, 11]. Although anoikis uses core apoptotic execution machinery, including mitochondrial dysfunction, Bcl‐2 family regulation, caspase activation, and DNA fragmentation, it is conceptually distinct from general apoptosis because its initiating signal is adhesion loss rather than DNA damage, cytokine stimulation, oncogene activation, or direct cytotoxic stress. Thus, anoikis represents an adhesion‐dependent surveillance mechanism that maintains epithelial tissue architecture by eliminating cells that detach from their proper microenvironment [8, 12, 13].

This distinction is particularly important in cancer biology. During metastasis, tumor cells detach from the primary tumor, survive without normal ECM support, invade surrounding tissues, enter lymphatic or blood vessels, withstand transit‐associated stress, and colonize secondary sites [14, 15]. Under physiological conditions, detached epithelial cells should undergo anoikis; however, malignant cells that acquire anoikis resistance can survive in suspension or in inappropriate ECM niches, thereby gaining a key prerequisite for dissemination and metastatic colonization [16, 17]. In GC, this process is highly relevant because tumor cell detachment and survival in the peritoneal cavity are central events in peritoneal dissemination, one of the most clinically challenging metastatic patterns of GC. Experimental evidence further supports this concept, as enhancement of caspase‐8‐mediated anoikis suppresses peritoneal dissemination of human GC cells [6].

Mechanistically, anoikis resistance in GC should be viewed not simply as reduced apoptosis, but as a coordinated metastatic survival program [8, 16, 17, 18, 19]. Detached GC cells can evade anoikis by restoring survival signals normally provided by adhesion, including integrin/focal adhesion kinase (FAK)/Src, phosphoinositide 3‐kinase‐protein kinase B/AKT serine‐threonine kinase (PI3K‐AKT), brain‐derived neurotrophic factor (BDNF) mitogen‐activated protein kinase‐extracellular signal‐regulated kinase (MEK‐ERK), nuclear factor kappa B (NF‐κB), Wnt/β‐catenin, yes‐associated protein (YAP)/TEA domain transcription factor (TEAD), BDNF/tropomyosin receptor kinase B (TrkB), and autophagy‐related pathways [18, 19, 20, 21, 22, 23, 24, 25]. These signaling networks allow tumor cells to maintain mitochondrial integrity, suppress caspase activation, remodel the cytoskeleton, undergo epithelial–mesenchymal transition (EMT), adapt metabolically, and survive during local invasion, lymphovascular spread, and peritoneal seeding [7, 16, 17, 21, 22, 24, 25, 26]. Therefore, anoikis resistance links tumor initiation and progression to metastatic dissemination and therapeutic resistance, making it a biologically and clinically important focus in GC [8, 16, 17].

To provide a clearer mechanistic framework, this review conceptualizes anoikis in GC as a three‐step biological program: initiation, execution, and evasion [8, 12, 16, 17, 18, 19]. Initiation begins when gastric epithelial or cancer cells lose appropriate ECM and cell–cell adhesion, especially integrin–ECM and cadherin‐mediated contacts, thereby disrupting focal adhesion and cytoskeletal survival signals [18, 19, 23]. Execution refers to the subsequent convergence of adhesion loss on mitochondrial and death‐receptor apoptotic machinery, including Bcl‐2 family remodeling, caspase‐8/caspase‐9 activation, and downstream executioner caspases [27, 28]. Evasion occurs when GC cells replace lost adhesion‐dependent survival cues with oncogenic compensatory circuits, particularly integrin/FAK/Src, PI3K‐AKT, MEK‐ERK, Wnt/β‐catenin, YAP/TEAD, and BDNF/TrkB signaling [8, 12, 16, 17, 18, 19, 20, 21, 22, 23, 24, 25]. Therefore, throughout this review, apoptosis‐related molecules are discussed only in relation to their detachment‐dependent activation or suppression, whereas anoikis‐specific mechanisms are defined by their dependence on altered ECM sensing, focal adhesion signaling, cytoskeletal tension, and anchorage‐independent survival. This framework helps integrate individual GC‐associated mediators into a coherent model of metastatic survival rather than treating them as isolated findings.

## 2 Molecular Mechanisms of Anoikis: From Adhesion Loss to Apoptotic Execution

Anoikis is often described as a subtype of apoptosis, but mechanistically it should not be introduced only through the classical intrinsic and extrinsic apoptotic pathways. The defining feature of anoikis is that the upstream death signal originates from loss of appropriate cell–ECM attachment or disruption of normal cell–cell adhesion [29]. Therefore, anoikis contains two mechanistic layers: an anoikis‐specific sensing layer, which detects detachment, altered ECM engagement, focal adhesion disruption, and cytoskeletal imbalance; and a shared apoptotic execution layer, which uses mitochondrial permeabilization, death receptor signaling, Bcl‐2 family regulation, and caspase activation to complete cell death. This distinction is important because many molecules involved in anoikis, such as caspases and Bcl‐2 family proteins, are not unique to anoikis, whereas the adhesion‐dependent regulation of integrins, FAK/Src, PI3K‐AKT, Rho GTPases, YAP/transcriptional coactivator with PDZ‐binding motif (TAZ), and cytoskeletal tension specifies anoikis as a detachment‐induced death program [8, 12, 18, 19]. Figure 1 summarizes this stepwise framework, illustrating how loss of ECM–integrin or cell–cell adhesion disrupts focal adhesion and mechanotransduction signaling and subsequently converges on mitochondrial and death‐receptor apoptotic execution.

**Figure 1:**
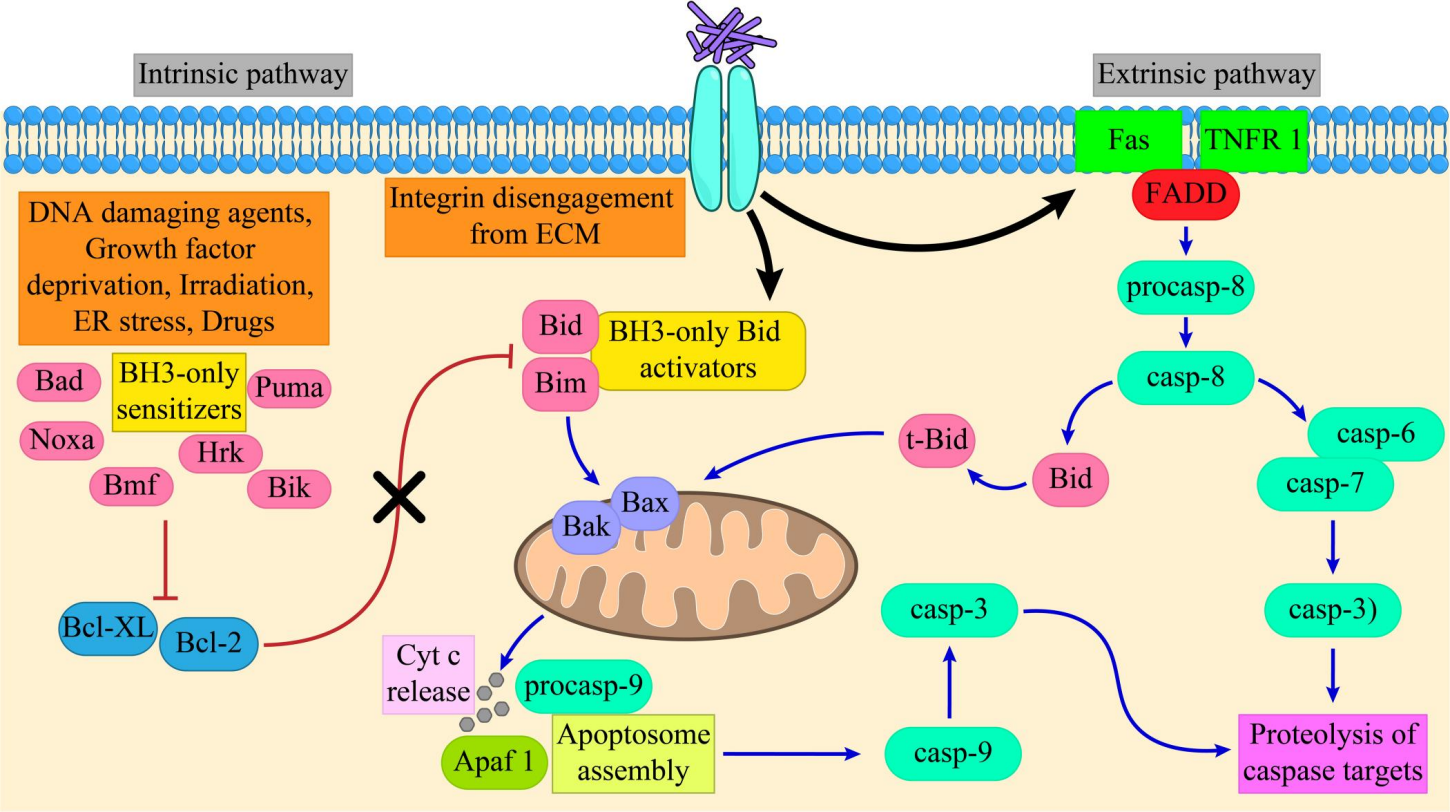
Stepwise molecular framework of anoikis. Anoikis begins with loss of appropriate ECM–integrin attachment or disruption of normal cell–cell adhesion. This initiates focal adhesion disassembly, reduced FAK/Src activity, altered PI3K–AKT and MEK‐ERK survival signaling, cytoskeletal remodeling, and mechanotransduction changes involving Rho‐family GTPases and Hippo–YAP/TAZ signaling. These anoikis‐specific upstream events are coupled to shared apoptotic execution machinery. In the intrinsic pathway, BH3‐only proteins such as Bim, Bmf, and Bid regulate Bax/Bak activation, mitochondrial outer membrane permeabilization, cytochrome c release, apoptosome formation, caspase‐9 activation, and executioner caspase cleavage. In the extrinsic pathway, death receptors such as Fas, TNFR1, DR4, and DR5 recruit FADD and activate caspase‐8, which can directly activate executioner caspases or amplify mitochondrial apoptosis through Bid. Thus, anoikis is specified by adhesion‐loss sensing and mechanotransduction, whereas caspases and Bcl‐2 family proteins constitute the shared apoptotic execution machinery. Apaf 1: Apoptotic protease activating factor 1, Casp: caspase, Cyt C: cytochrome c, ECM: extracellular matrix, ER: endoplasmic reticulum, FADD: Fas‐associated death domain protein, TNFR: tumor necrosis factor receptor.

### 2.1 ECM Detachment Sensing and Focal Adhesion Signaling

In adherent epithelial cells, survival is actively maintained by integrin‐mediated adhesion to the ECM and by cadherin‐dependent cell–cell contacts. Integrins cluster at focal adhesions and recruit FAK, Src family kinases, paxillin, talin, vinculin, and other scaffold proteins that connect the ECM to the actin cytoskeleton. This adhesion complex is not merely structural; it acts as a survival signaling platform by activating PI3K‐AKT, Ras‐MEK‐ERK, and other downstream pathways that suppress apoptotic priming [30]. When epithelial cells detach from the ECM or attach to an inappropriate matrix, integrin clustering and focal adhesion architecture are disrupted. This loss of adhesion weakens FAK/Src‐dependent survival signaling and removes tonic PI3K‐AKT and ERK inputs that normally restrain pro‐apoptotic proteins. Thus, ECM detachment sensing represents the first anoikis‐specific step, upstream of mitochondrial or death receptor apoptosis [12, 18, 19, 31].

In cancer cells, including GC cells, this sensing step can be bypassed by integrin switching, altered ECM remodeling, growth factor receptor cooperation, or constitutive activation of downstream survival pathways [32]. For example, integrin and epidermal growth factor receptor (EGFR) signaling can cooperate to suppress the pro‐apoptotic Bcl‐2 homology 3 (BH3)‐only protein Bim, thereby preventing detachment‐induced apoptosis. This illustrates why anoikis resistance is not simply “less apoptosis,” but rather a rewiring of adhesion‐dependent survival signaling after detachment [20].

### 2.2 Cytoskeletal Disruption and Mechanotransduction

ECM detachment also causes profound changes in cell shape, actin organization, cytoskeletal tension, and mechanotransduction. In adherent cells, actin stress fibers and focal adhesions transmit mechanical information from the ECM to intracellular signaling networks. Detachment disrupts this mechanical continuity, leading to changes in Rho‐family GTPase activity, actomyosin contractility, and cytoskeletal architecture. These changes regulate both survival signaling and apoptotic sensitivity [33]. Therefore, cytoskeletal remodeling should be considered a central anoikis‐specific regulatory event rather than a secondary morphological consequence.

A major mechanotransduction pathway involved in detachment‐induced cell death is the Hippo–YAP/TAZ axis. In non‐transformed cells, cell detachment and cytoskeletal reorganization activate Hippo pathway kinases, resulting in phosphorylation and inhibition of YAP/TAZ. Because YAP/TAZ promote transcriptional programs related to proliferation, survival, and stemness, their inactivation facilitates anoikis [23]. Conversely, cancer cells with deregulated YAP/TAZ signaling can maintain survival after detachment, supporting anchorage‐independent growth and metastatic colonization. This is particularly relevant to GC because YAP1 is enriched in gastric adenocarcinoma peritoneal metastasis cells and YAP1 inhibition attenuates peritoneal colonization; more directly, glucose‐regulated protein 94 (GRP94)‐driven anoikis resistance in GC has been mechanistically linked to YAP/TEAD1 activation [21, 22].

### 2.3 Coupling ECM Detachment to Intrinsic Mitochondrial Apoptosis

After detachment sensing and mechanotransduction changes, anoikis signals are transmitted to the intrinsic mitochondrial apoptotic pathway. This step is mediated mainly by Bcl‐2 family proteins. The Bcl‐2 family includes anti‐apoptotic members such as Bcl‐2, Bcl‐xL, and myeloid cell leukemia 1 (Mcl‐1); multidomain pro‐apoptotic proteins such as Bax, Bak, and Bok; and BH3‐only proteins such as Bim, Bmf, Bad, Bid, Bik, Puma, Noxa, and Hrk. During anoikis, the loss of adhesion‐dependent PI3K‐AKT and ERK signaling favors induction or activation of BH3‐only proteins, especially Bim and Bmf [34]. These proteins either directly activate Bax/Bak or neutralize anti‐apoptotic Bcl‐2 family proteins, leading to mitochondrial outer membrane permeabilization, cytochrome c release, apoptosome formation, caspase‐9 activation, and downstream activation of executioner caspases such as caspase‐3 and caspase‐7 [20, 27, 28].

This intrinsic pathway is shared with other forms of apoptosis, but its regulation in anoikis is specified by detachment‐dependent upstream cues. For example, in DNA damage‐induced apoptosis, mitochondrial permeabilization is initiated primarily by genotoxic stress and p53‐related pathways. In anoikis, by contrast, mitochondrial apoptosis is triggered by loss of integrin/FAK/Src, EGFR–integrin, PI3K‐AKT, and ERK‐mediated survival signals [34]. Thus, the mitochondrial machinery is general apoptotic machinery, whereas its activation through adhesion loss is anoikis‐specific.

### 2.4 Death Receptor Signaling in Anoikis

In addition to mitochondrial apoptosis, death receptor signaling can contribute to anoikis execution. The extrinsic pathway is mediated by members of the tumor necrosis factor receptor superfamily, including Fas, TNFR1, and TNF‐related apoptosis‐inducing ligand (TRAIL) receptors death receptor (DR)4 and DR5. Ligand binding or receptor activation promotes assembly of the death‐inducing signaling complex (DISC), recruitment of Fas‐associated death domain protein (FADD), and activation of caspase‐8. Activated caspase‐8 can directly cleave and activate downstream executioner caspases or amplify mitochondrial apoptosis through Bid cleavage and mitochondrial engagement [27, 34].

However, death receptor signaling should be interpreted carefully in the context of anoikis. Fas, TNFR, TRAIL receptors, FADD, and caspase‐8 are not anoikis‐specific molecules, because they also mediate classical extrinsic apoptosis in many biological settings. Their relevance to anoikis depends on detachment‐associated activation, sensitization, or cooperation with mitochondrial apoptosis. In GC, this link is clinically relevant because experimental restoration of caspase‐8 activity has been shown to augment anoikis and suppress peritoneal dissemination of human GC cells [6]. Therefore, caspase‐8 represents an apoptotic execution node that becomes functionally connected to anoikis when activated in response to anchorage loss.

### 2.5 Key Regulatory Nodes Specifying Anoikis

The anoikis program is controlled by several regulatory nodes that connect adhesion loss to apoptotic execution. First, the integrin/FAK/Src axis functions as the proximal adhesion‐sensing module. When ECM engagement is appropriate, this module sustains survival through PI3K‐AKT and ERK signaling; when ECM attachment is lost, suppression of this axis permits apoptotic priming [18, 19, 20]. Second, PI3K‐AKT and MEK‐ERK act as central pro‐survival pathways that restrain BH3‐only proteins and preserve mitochondrial integrity, thereby limiting detachment‐induced mitochondrial apoptosis [20, 27, 28]. Third, Rho‐family GTPases and cytoskeletal regulators translate mechanical changes into biochemical death or survival signals, linking cell shape and cytoskeletal tension to anoikis sensitivity [23]. Fourth, the Hippo‐YAP/TAZ pathway connects cytoskeletal organization and mechanical state to transcriptional control of survival, and deregulated YAP/TEAD signaling has also been implicated in GC peritoneal metastasis and anoikis resistance [21, 22, 23]. Fifth, Bcl‐2 family proteins and caspase‐dependent pathways form the apoptotic execution layer that determines whether detachment signals culminate in mitochondrial outer membrane permeabilization and executioner caspase activation [27, 28].

These nodes clarify how anoikis differs from apoptosis in general. General apoptosis can be initiated by diverse stimuli such as DNA damage, ER stress, cytokine withdrawal, immune‐mediated death receptor activation, or cytotoxic therapy. Anoikis, in contrast, is initiated by loss of proper adhesion and is specified by altered integrin engagement, focal adhesion disassembly, cytoskeletal remodeling, mechanotransduction changes, and loss or rewiring of adhesion‐dependent survival signals [8, 12, 18, 19, 23]. Therefore, the caspase cascade and Bcl‐2 family proteins represent the shared execution machinery, whereas integrins, FAK/Src, PI3K‐AKT, Rho GTPases, YAP/TAZ, and ECM/cytoskeletal context define the anoikis‐specific regulatory layer [8, 12, 18, 19, 23, 27, 28].

### 2.6 Relevance of the Mechanistic Framework to GC

This framework is directly relevant to GC progression because local invasion and peritoneal dissemination require GC cells to detach, survive without basement‐membrane support, resist suspension‐induced stress, and colonize serosal or distant sites. These steps require evasion of anoikis [7]. Therefore, GC‐associated anoikis resistance can be understood as a progressive shift from adhesion‐dependent survival to anchorage‐independent survival. In this process, detached GC cells maintain or reactivate survival pathways such as integrin/FAK/Src, PI3K‐AKT, MEK‐ERK, NF‐κB, Wnt/β‐catenin, YAP/TEAD, BDNF/TrkB, and autophagy‐related signaling. The following sections discuss how GC‐specific mediators exploit these nodes to suppress anoikis, promote metastatic dissemination, and create therapeutic vulnerabilities [17].

Before discussing GC‐specific anoikis‐resistance mediators, it is important to place anoikis within its physiological and tumor‐suppressive context. In normal epithelial tissues, including the gastric mucosa, epithelial cells depend on appropriate basement‐membrane ECM engagement and cell–cell adhesion for positional survival signals. Cells that lose these spatial cues are normally eliminated through detachment‐induced apoptosis, thereby preserving epithelial architecture and preventing displaced, damaged, or potentially dysplastic cells from surviving in inappropriate microenvironments [8, 9, 12]. In this regard, anoikis functions as an intrinsic tumor‐suppressive barrier: cells that acquire early oncogenic alterations but remain dependent on integrin‐ and cadherin‐mediated anchorage are removed before they can expand, invade, or colonize ectopic niches [16, 17]. During gastric tumorigenesis, this barrier is progressively disrupted through altered ECM sensing, focal‐adhesion remodeling, cytoskeletal reprogramming, EMT, hypoxia‐driven adaptation, and growth‐factor‐dependent survival signaling. As a result, detached GC cells can replace lost adhesion‐derived survival cues with compensatory circuits such as integrin/FAK/Src, EGFR–integrin subunit beta 1 (ITGB1), PI3K‐AKT, MEK‐ERK, NF‐κB, Wnt/β‐catenin, YAP/TEAD, BDNF/TrkB, and protective autophagy. These mechanisms suppress mitochondrial and death‐receptor execution of anoikis, permit survival in suspension or ascitic fluid, and facilitate local invasion, lymphovascular dissemination, peritoneal metastasis, and poor clinical outcome [6, 22, 26, 35, 36, 37]. Therefore, anoikis resistance in GC should be interpreted as a coordinated metastatic survival program rather than as an isolated reduction in apoptosis.

## 3 Anoikis Resistance in GC

GC metastatic progression depends critically on anoikis resistance, although the underlying mechanisms remain incompletely defined. In GC, anoikis resistance can be organized into interconnected modules rather than a single linear pathway. Upstream adhesion‐related mechanisms include hypoxia‐driven suppression of α5‐integrin, transcription factor 7‐like 2 (TCF7L2)‐mediated transcriptional activation of plasminogen activator, urokinase receptor (PLAUR/uPAR), Ras homolog family member A (RHOA)‐ associated disruption of adhesion signaling, and cytoskeletal remodeling through myosin heavy chain 9 (MYH9)/catenin beta 1 (CTNNB1) signaling [26, 36, 38, 39]. These upstream changes converge on intracellular survival and transcriptional hubs, including EGFR–ITGB1–Src, PI3K‐AKT, Wnt/β‐catenin, YAP/TEAD, NF‐κB, BDNF/TrkB, and autophagy‐related programs [22, 24, 25, 40, 41]. This framework provides a clearer structure for interpreting the GC‐specific mediators reviewed below.

### 3.1 Upstream Adhesion, ECM, and Cytoskeletal Regulators of Anoikis Resistance in GC

Hypoxia‐inducible factor 1‐alpha (HIF‐1α) provides a direct mechanistic link between the hypoxic tumor microenvironment and detachment survival in GC. In GC cells, HIF‐1α mediates anoikis resistance by suppressing α5‐integrin; conversely, restoration of α5‐integrin in HIF‐1α‐deficient cells increases anoikis sensitivity, indicating that hypoxia promotes metastatic competence partly by rewiring integrin‐dependent adhesion signaling rather than only by enhancing proliferation or invasion [35]. Consistently, HIF‐1α expression at the invasive tumor edge has been associated with metastatic potential in human GC [38]. PLAUR contributes to GC progression by promoting tumor‐cell migration and survival. *Zhang* et al. established that PLAUR enhances the anoikis resistance and the metastasis of GC cells, identifying transcription factor‐7 like 2 (TCF7L2) as a significant transcriptional regulator of PLAUR. They further showed that TCF7L2 is overexpressed in GC and promotes both anoikis resistance and metastatic potential. In addition, their research indicated that TCF7L2 transcriptionally activates PLAUR and suggested that TCF7L2 serves as an independent prognostic factor for unfavorable outcomes in GC patients. These findings identify the TCF7L2–PLAUR axis as a potential therapeutic target for GC metastasis (Figure 2) [26]. According to whole‐genome sequencing studies, the most disrupted route in GC is the adherens junction and focal adhesion, while RHOA mutations in diffuse‐type cancers encouraged anoikis escape. RHOA plays a significant role, indicating that anoikis could be a viable target throughout GC [39]. MYH9 was shown to be elevated in metastatic GC tissues and correlated with a poor outcome in GC patients. MYH9 has been found to modulate CTNNB1 expression, hence inducing anoikis resistance and facilitating GC metastasis [36]. This finding links cytoskeletal remodeling to transcriptional survival signaling: nuclear MYH9 increases CTNNB1 transcription, activates Wnt/β‐catenin signaling, promotes anoikis resistance and peritoneal metastasis, and can be pharmacologically suppressed by staurosporine in preclinical GC models [36]. Integrin subunit beta like 1 (ITGBL1), which encodes a protein featuring 10 epidermal growth factor (EGF) repeat domains characteristic of integrins, was first identified from a cDNA library obtained from osteoblasts in 1999 [42]. Growth differentiation factor 15 (GDF15) was found to be an important component in GC anoikis resistance by *Gao* et al., using quantitative proteomics. Research has shown that GDF15 can help GC cells acquire anoikis resistance by increasing protective autophagy. Additionally, activating transcription factor 2 (ATF2) was identified as a crucial regulator of GDF15 production and a transcriptional activator of GDF15 by a DNA pull‐down experiment [37]. P600 (a 600‐kDa protein) was extracted from cervical cancer cells and identified as a retinoblastoma‐associated binding protein [43, 44]. Expression of p600 mRNA was markedly elevated in GC cells compared to normal mucosal cells, as reported by *Sakai* et al. [45]. The invasion assay demonstrated that invasiveness was markedly diminished by the suppression of p600 [45]. S100 calcium‐binding protein A4 (S100A4) exerts a negative regulatory effect on anoikis. Inhibition of S100A4 decreases the expression of α5 and αv integrins, indicating that S100A4 facilitates anoikis resistance through the modulation of αv and α5 integrin levels, hence contributing to metastasis in GC cells [46].

**Figure 2:**
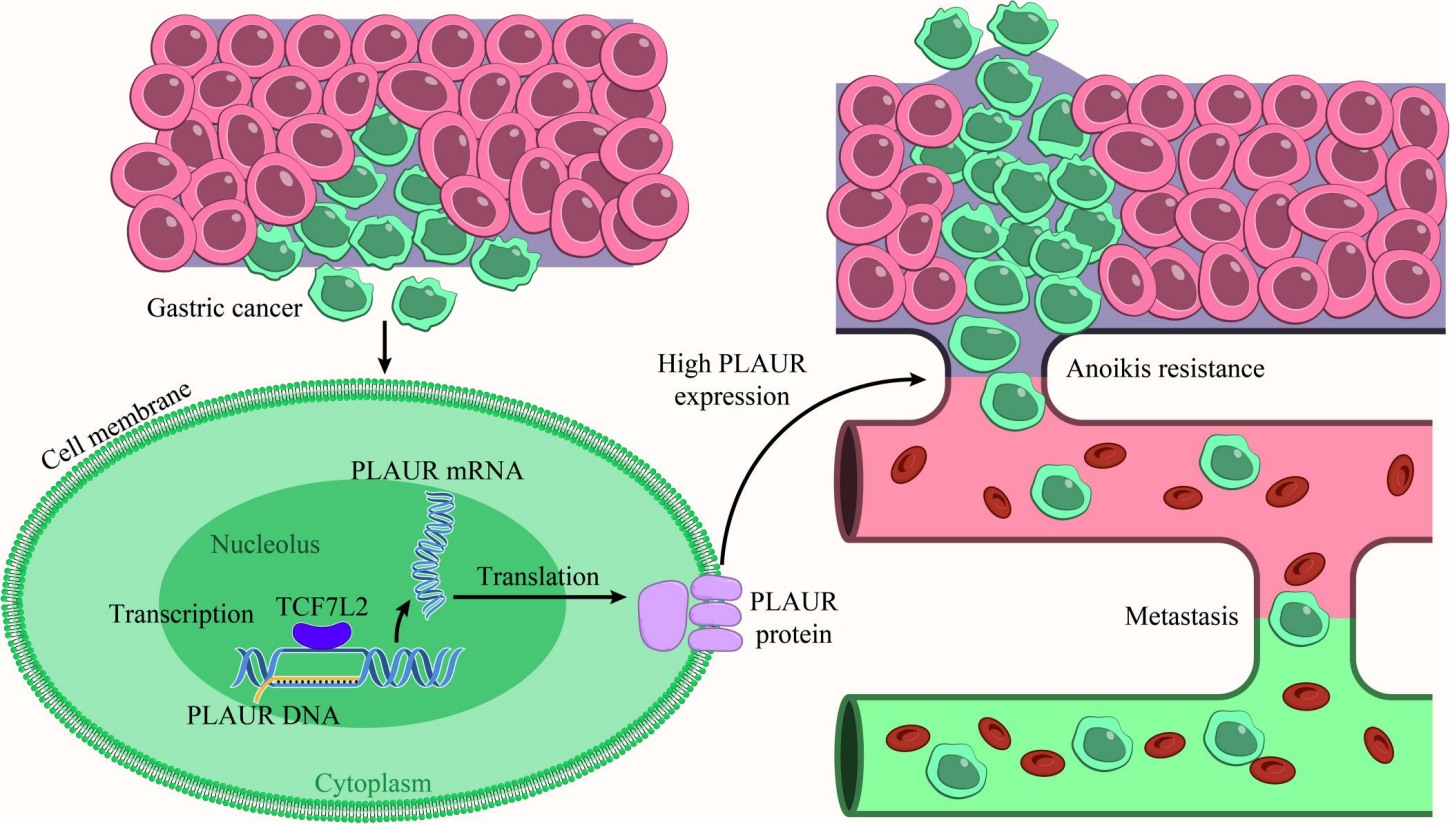
TCF7L2‐mediated transcriptional activation of PLAUR promotes GC metastasis. The PLAUR plays a critical role in the malignant progression of GC by enhancing cell migration and survival. The transcription factor TCF7L2 directly binds to the PLAUR promoter region, transcriptionally activating its expression. Elevated TCF7L2 levels in GC cells were found to increase anoikis resistance and promote metastatic dissemination. TCF7L2 and PLAUR are significantly overexpressed in GC tissues and that high TCF7L2 expression correlates with poor patient prognosis. These findings indicate that the TCF7L2–PLAUR regulatory axis contributes to GC progression and represents a promising therapeutic target for inhibiting metastasis. GC: Gastric cancer, PLAUR: plasminogen activator receptor, TCF7L2: transcription factor 7 like 2.

### 3.2 Signaling Pathways and Regulatory Networks Involved in Anoikis Resistance in GC

#### 3.2.1 NF‐κB‐related Signaling

Deleted in breast cancer 1 (DBC1) is overexpressed in several malignancies and is associated with poor prognosis. Research has demonstrated that DBC1 is overexpressed in GC and is significantly linked to the tumor, node, and metastasis (TNM) staging and lymph node metastasis. DBC1 expression is associated with anoikis resistance in GC cells, a key feature of metastatic progression. Moreover, the findings indicated that the regulation of anoikis resistance by DBC1 in GC cells involved the IκB kinase beta (IKKβ)/NF‐κB signaling pathway [47].

#### 3.2.2 BDNF/TrkB and TRIM69/PRKCD/BDNF Signaling

BDNF activates the high‐affinity TrkB receptor and has direct clinical and experimental support in GC progression. In GC samples, increased BDNF expression at the invasive front correlates with disease progression, poor prognosis, lymph node metastasis, and peritoneal dissemination; functionally, recombinant BDNF promotes proliferation, migration, invasion, and anoikis resistance, whereas Trk inhibition suppresses these effects and reduces peritoneal dissemination in nude mice [25]. More recent work connects this pathway to tripartite motif‐containing 69 (TRIM69)/protein kinase C delta (PRKCD) regulation: TRIM69 is reduced in anoikis‐resistant GC cells, and its restoration suppresses anoikis resistance and metastasis by promoting K48‐linked polyubiquitination of PRKCD, thereby reducing PRKCD‐dependent BDNF production; rescue with PRKCD or BDNF reverses the anti‐metastatic effect of TRIM69 [41].

#### 3.2.3 PI3K‐AKT/ECM‐related Signaling: ITGBL1/AKT/FBLN2 and CAV1/EGFR/ITGB1

ITGBL1 has direct evidence as an anoikis‐resistance mediator in GC. ITGBL1 overexpression enhances anoikis resistance and metastatic potential, whereas ITGBL1 knockdown suppresses these processes in vitro and in vivo. Mechanistically, ITGBL1 promotes anoikis resistance through the AKT/fibulin 2 (FBLN2) axis, and inhibition of AKT/FBLN2 signaling reverses the pro‐metastatic and anti‐anoikis effects of ITGBL1. Clinically, high ITGBL1 expression is associated with worse prognosis, particularly when combined with reduced FBLN2 expression [40]. Because FBLN2 may also exert tumor‐suppressive effects in GC, the ITGBL1/FBLN2 relationship should be interpreted as a context‐dependent ECM‐survival axis rather than a simple one‐directional pathway [48].

Caveolin 1 (CAV1) should be discussed within the PI3K‐AKT/ECM‐related signaling module because its anoikis‐related function in GC is mediated through EGFR–ITGB1–Src signaling rather than through YAP/TEAD. In detached SGC‐7901 cells, CAV1 promotes anchorage‐independent growth and anoikis resistance by enhancing Src‐dependent EGFR–ITGB1 signaling, followed by activation of MEK‐ERK and PI3K‐AKT pathways [24]. Thus, CAV1 provides a mechanistic bridge between altered membrane organization, integrin/growth‐factor receptor cooperation, and downstream survival signaling after detachment. However, its interpretation should remain context‐dependent because tumor‐cell and stromal CAV1 may have different prognostic implications in GC.

#### 3.2.4 YAP/TEAD Signaling: GRP94/YAP–TEAD1

Shi et al. have underscored the role of GRP94 in facilitating anoikis resistance and peritoneal metastasis in GC. The study revealed that the overexpression of GRP94, which is associated with an unfavorable prognosis, may be attributed to the interactions between specificity protein 1 (SP1) and the GRP94 promoter. An elevation in GRP94 levels resulted in heightened anoikis resistance and metastatic potential in GC cells. Decreasing GRP94 exhibited contrasting effects, possibly via the inhibition of the YAP/TEAD1 pathway, characterized by increased YAP phosphorylation and reduced TEAD1 levels. The inhibition of YAP mitigated the impacts of GRP94 on anoikis resistance and metastasis, whereas the activation of YAP counteracted the effects resulting from GRP94 depletion. Experiments on animals confirmed the role of GRP94 in the peritoneal metastasis of GC [22]. Relative to other candidates, GRP94/YAP–TEAD1 possesses robust mechanistic depth (SP1‐driven transcription; YAP phosphorylation changes; pharmacologic rescue) and in vivo peritoneal validation, supporting a higher translational priority [22]. Together, these findings position GRP94/YAP‐TEAD1 as one of the better‐supported anoikis‐resistance axes in GC because it is supported by transcriptional regulation, pathway rescue, anoikis assays, and in vivo peritoneal metastasis validation [22].

#### 3.2.5 Wnt/β‐catenin and EMT‐Related Signaling: MYH9/CTNNB1 and CCT5

Chaperonin‐containing TCP1 subunit 5 (CCT5), a component of the TCP1 ring complex/chaperonin‐containing TCP1 (TRiC/CCT) chaperonin complex, has been implicated in GC progression and lymph node metastasis. CCT5 is upregulated in lymph node metastatic GC and is associated with poor prognosis. Functionally, CCT5 promotes proliferation, invasion, anoikis resistance, lymphangiogenesis, xenograft growth, and popliteal lymph node metastasis in GC models [49]. Mechanistically, CCT5 interacts with the cytoplasmic domain of E‐cadherin and disrupts the E‐cadherin/β‐catenin complex, thereby facilitating β‐catenin nuclear translocation, Wnt/β‐catenin activation, and EMT. Within the anoikis‐resistance framework, CCT5 can therefore be positioned as a Wnt/β‐catenin–EMT amplifier that weakens adhesion‐dependent epithelial constraints and promotes anchorage‐independent behavior. Nevertheless, because much of the available evidence emphasizes lymph node metastasis rather than peritoneal dissemination, future studies should validate whether CCT5 directly regulates detachment‐induced apoptosis under suspension conditions and whether its anoikis‐related effects are reversible by β‐catenin inhibition or E‐cadherin restoration [49].

Taken together, the strongest mechanistic support currently exists for the GRP94/YAP–TEAD1, MYH9/CTNNB1, and ITGBL1/AKT/FBLN2 axes because these pathways combine gain‐ and loss‐of‐function experiments, pathway rescue, clinical association, and in vivo metastasis or peritoneal dissemination models [22, 36, 40]. BDNF/TrkB and TRIM69/PRKCD/BDNF also have direct anoikis‐resistance evidence but require further biomarker‐defined validation, whereas DBC1/NF‐κB, HIF‐1α/α5‐integrin, and CAV1/EGFR–ITGB1 should be interpreted as context‐ or model‐dependent regulators rather than uniformly dominant drivers [24, 25, 35, 41].

#### 3.2.6 miRNA‐Mediated Regulation of Anoikis‐Resistance Signaling

microRNAs (miRNAs) should be integrated into the signaling framework of anoikis resistance because their effects converge on survival, EMT, Wnt/β‐catenin, C‐X‐C motif chemokine receptor 4 (CXCR4), sirtuin 1 (SIRT1), E2F, and uPAR‐related pathways. In anoikis‐resistant SGC‐7901 cells, miR‐497 is downregulated, whereas restoration of miR‐497 suppresses proliferation, invasion, migration, tumor growth, EMT marker switching, and Wnt/β‐catenin activation [50]. Similarly, miR‐204‐5p suppresses lymph node metastasis through regulation of the CXCL12/CXCR4 axis, while miR‐204 also reverses SIRT1‐induced EMT, invasion, and anoikis resistance in GC cells [51, 52].

In contrast, miR‐376c promotes anchorage‐independent oncogenic behavior by suppressing AT‐rich interaction domain 4 A (ARID4A) and enhancing gastric carcinoma cell proliferation, migration, and anchorage‐independent growth [53]. The miR‐335‐5p/uPAR axis provides another link between non‐coding RNA regulation and extracellular‐matrix remodeling: miR‐335‐5p is reduced in GC, correlates with lymph node metastasis and poor prognosis, and suppresses invasion and anchorage‐independent growth partly through PLAUR/uPAR‐related signaling. Because urokinase‐type plasminogen activator (uPA)/uPAR signaling protects cells from anoikis by increasing BCL‐xL expression, loss of miR‐335‐5p may reinforce the same pro‐survival circuits activated by PLAUR overexpression.

Collectively, these findings indicate that anoikis resistance in GC is not driven by a single linear pathway but by a convergent signaling network centered on adhesion‐sensitive survival hubs. Integrin/FAK/Src signaling represents the proximal sensor module that links ECM status to PI3K‐AKT and ERK‐dependent survival. PI3K‐AKT functions as a central anti‐anoikis hub by sustaining pro‐survival phosphorylation events, supporting Bcl‐2 family‐mediated mitochondrial protection, and cooperating with EMT and metabolic adaptation. Wnt/β‐catenin and YAP/TEAD act as transcriptional output modules that stabilize anchorage‐independent growth, invasiveness, and peritoneal colonization. In parallel, BDNF/TrkB, NF‐κB, and autophagy‐related pathways provide context‐specific reinforcement of detached‐cell survival. Therefore, therapeutic targeting of anoikis resistance in GC is likely to require biomarker‐guided interruption of upstream adhesion signaling together with downstream survival and transcriptional programs, rather than inhibition of isolated apoptotic effectors alone [18, 19, 22, 31, 36, 40, 41].

### 3.3 Comparative Appraisal of Prioritized Anoikis Resistance Pathways in GC

Here, we comparatively evaluated key anoikis resistance axes with the highest translational interest in GC, namely ITGBL1/AKT–FBLN2, GRP94/YAP–TEAD1, and BDNF/TrkB, against other frequently reported mediators. We scored each pathway by: mechanistic depth (gain/loss‐of‐function ± rescue), in vivo validation (metastasis/anoikis models), clinical association (TNM/overall survival (OS)), and reproducibility/consistency across models. Table 1 summarizes the head‐to‐head evidence and outstanding conflicts/limitations, including in vivo support where available.

**Table 1:** Comparative summary of key anoikis resistance pathways in GC.

Pathway/Axis	Key players	Primary experimental evidence (mechanism)	In vivo validation	Clinical association	Evidence strength	Conflicts/notes	Ref.
ITGBL1 → AKT → FBLN2	ITGBL1, AKT, FBLN2	Gain/loss ± rescue: ITGBL1 overexpression ↑anoikis resistance & metastasis; AKT/FBLN2 inhibition reverses phenotype	xenograft/peritoneal models	↑TNM, distant mets, worse OS	High	FBLN2 shows tumor‐suppressor features in GC, context matters	[40]
GRP94 → YAP/TEAD1	GRP94, YAP, TEAD1, SP1	Overexpression ↑anoikis resistance & peritoneal mets; YAP inhibition rescues; SP1 drives GRP94 promoter	peritoneal metastasis models	Linked to poor prognosis	High	—	[22]
BDNF → TrkB	BDNF, TrkB	rhBDNF ↑proliferation/migration & anoikis resistance; Trk inhibitor (K252a) blocks; TRIM69 suppresses BDNF via PRKCD	peritoneal dissemination in nude mice; inhibitor blocks	Poor prognosis; metastasis associations	Moderate–High	Many studies pre‐2015; limited genetic rescue in vivo	[25]
CAV1 → EGFR/ITGB1 (Src–MEK–ERK; PI3K–AKT)	CAV1, EGFR, ITGB1	CAV1 ↑anchorage‐independent growth & anoikis resistance via EGFR–integrin‐β1/Src axes	Mixed in vivo	Prognostic data conflicted (tumor vs stromal CAV1)	Mixed/Context‐dependent	Acts as oncogene or tumor‐suppressor depending on context; stromal CAV1 low = poor prognosis	[24, 54]
DBC1 (CCAR2) → IKKβ/NF‐κB	DBC1/CCAR2, IKKβ, NF‐κB	In vitro: DBC1 ↑ NF‐κB activity → ↑anoikis resistance	No GC in vivo yet	Some expression–stage links	Preliminary	CCAR2 shows dual roles across cancers	[47]
HIF‐1α ⟂ α5‐integrin	HIF‐1α, ITGA5	HIF‐1α suppresses α5‐integrin → mediates anoikis resistance (detachment models)	No GC in vivo yet	—	Preliminary	Integrin‐context/ECM‐dependence complicates generalization	[35, 38]
MYH9 → β‐catenin (CTNNB1)	MYH9, β‐catenin	Nuclear MYH9 drives CTNNB1 transcription → ↑anoikis resistance; staurosporine inhibits axis	orthotopic xenograft & transgenic	↑metastasis; poorer outcomes	High	—	[36]

Abbreviations: AKT, Protein kinase B (PKB); BDNF, Brain‐Derived Neurotrophic Factor; CAV1, Caveolin‐1; CCAR2, Cell Cycle and Apoptosis Regulator 2 (DBC1); CTNNB1, Catenin Beta‐1 (β‐Catenin); DBC1, Deleted in Breast Cancer 1 (CCAR2); ECM, Extracellular Matrix; EGFR, Epidermal Growth Factor Receptor; ERK, Extracellular Signal‐Regulated Kinase (MAPK1/3); FBLN2, Fibulin‐2; GC, Gastric Cancer; GRP94, Glucose‐Regulated Protein 94 (HSP90B1, endoplasmin/gp96); HIF‐1α, Hypoxia‐Inducible Factor‐1 alpha (HIF1A); IKKβ, IκB Kinase β subunit (IKBKB); ITGA5, Integrin Alpha‐5 (CD49e); ITGB1, Integrin Beta‐1 (CD29); ITGBL1, Integrin Beta‐Like 1 (TIED); K252a, Trk inhibitor K252a (staurosporine analog); MEK, MAPK/ERK Kinase (MAP2K1/2); MYH9, Myosin Heavy Chain 9 (non‐muscle myosin IIA heavy chain); mets, Metastases (distant metastases); NF‐κB, Nuclear Factor kappa‐B; OS, Overall Survival; PI3K, Phosphoinositide 3‐Kinase; PRKCD, Protein Kinase C delta (PKC‐δ); rhBDNF, Recombinant Human BDNF; Src, Proto‐oncogene tyrosine‐protein kinase Src; SP1, Specificity Protein 1; TEAD1, TEA Domain Transcription Factor 1; TNM, Tumor‐Node‐Metastasis (staging system); TrkB, Tropomyosin Receptor Kinase B (NTRK2); YAP, Yes‐Associated Protein 1 (YAP1).

### 3.4 Conflicting or Low‐Reproducibility Findings

CAV1 shows context‐dependent behavior in GC. While several studies report CAV1‐driven anoikis resistance via EGFR–ITGB1 and downstream Src/ERK and PI3K–AKT signaling, stromal CAV1 loss has been associated with poorer outcomes, and meta‐analyses report heterogeneous prognostic effects. These discrepancies likely reflect tumor–stroma compartmentalization, disease stage, and methodological differences in quantification [55, 56, 57]. DBC1/cell cycle and apoptosis regulator 2 (CCAR2) promotes anoikis resistance in vitro via IKKβ/NF‐κB; however, CCAR2 can act as a tumor suppressor or promoter across contexts, warranting GC‐specific in vivo validation before prioritization [47].

Model‐dependence remains a key limitation. A number of GC studies, including some cited here, rely heavily on SGC‐7901 cells, which have been reported as HeLa‐contaminated/misidentified in International Cell Line Authentication Committee (ICLAC)/Cellosaurus. We therefore recommend cross‐validating key findings in authenticated GC lines (e.g., MKN45, AGS, HGC‐27, NUGC‐3) or patient‐derived organoids and explicitly stating short tandem repeat (STR) authentication in Methods [48]. Altogether, pathway inter‐dependencies create apparent contradictions: e.g., ITGBL1 promotes AKT activation while FBLN2 may exert tumor‐suppressive effects in GC; thus, ITGBL1's pro‐metastatic action may partially reflect repression of an anti‐metastatic ECM component [48].

### 3.5 Integrated Hierarchy of Anoikis‐Resistance Mechanisms in GC

To improve conceptual hierarchy, the GC‐associated mechanisms discussed above can be arranged into three interconnected levels. The first level comprises upstream adhesion and microenvironmental alterations, including disrupted integrin–ECM sensing, cadherin remodeling, hypoxia, ECM proteolysis, and cytoskeletal adaptation. This level includes mediators such as HIF‐1α/α5‐integrin, TCF7L2/PLAUR, RHOA, S100A4, p600, and CAV1/EGFR–ITGB1, which alter how GC cells perceive and respond to detachment stress [24, 26, 35]. The second level consists of intracellular survival and transcriptional hubs that replace lost adhesion‐dependent signals, including PI3K–AKT, MEK‐ERK, NF‐κB, Wnt/β‐catenin, YAP/TEAD, BDNF/TrkB, and autophagy‐related programs. Mechanistically strong examples include MYH9‐driven CTNNB1 transcription, ITGBL1‐mediated AKT/FBLN2 regulation, GRP94‐mediated YAP/TEAD1 activation, GDF15‐induced protective autophagy, and TRIM69/PRKCD‐dependent suppression of BDNF signaling [22, 36, 37, 40, 41]. The third level represents the execution phase, in which these upstream and intermediate alterations converge to preserve mitochondrial integrity, maintain anti‐apoptotic Bcl‐2 family signaling, limit caspase‐8/caspase‐9 activation, and enable survival of detached cells in suspension, ascites, or metastatic niches [6, 20, 27].

This hierarchical model also helps balance the evidence across individual findings. Pathways such as GRP94/YAP–TEAD1, MYH9/CTNNB1, and ITGBL1/AKT/FBLN2 currently have relatively strong mechanistic and/or in vivo support, whereas CAV1 and DBC1 require more context‐aware interpretation because their roles may depend on tumor‐cell versus stromal expression, cell‐line background, disease stage, and experimental model. CCT5 should be positioned as a Wnt/β‐catenin–EMT regulator with evidence for lymph node metastasis and xenograft growth, but not overinterpreted as a fully validated peritoneal anoikis‐resistance driver unless additional detachment‐specific rescue experiments are provided [49]. Therefore, rather than presenting all molecules with equal weight, GC anoikis resistance should be viewed as a layered network in which extracellular adhesion remodeling, intracellular survival hubs, miRNA‐mediated regulation, EMT/stemness, and apoptotic blockade cooperate to support metastatic dissemination and unfavorable clinical outcomes.

## 4 Therapeutic Targeting of Anoikis Resistance in GC: Biomarkers, Patient Stratification, and Clinical Translation

A major translational challenge in this field is to distinguish therapies that directly restore detachment‐induced cell death from therapies that broadly inhibit cancer growth but only indirectly intersect with anoikis‐related pathways. Therefore, therapeutic targeting of anoikis resistance in GC should not be presented simply as a list of anticancer agents. Instead, therapeutic strategies can be organized into two biologically distinct categories: (i) direct modulation of the anoikis program, including restoration of caspase‐dependent detachment‐induced death, disruption of integrin/EGFR‐mediated anchorage‐independent survival, inhibition of protective autophagy under suspension stress, or interference with cytoskeletal and RhoA‐dependent survival; and (ii) indirect targeting of anoikis‐resistance hubs, including FAK/Src, PI3K–AKT–mechanistic target of rapamycin (mTOR), MEK‐ERK, YAP/TEAD, Wnt/β‐catenin, BDNF/TrkB, and EMT‐associated programs. This distinction is important because many drugs discussed in GC therapy are not anoikis‐specific; their relevance to this review depends on whether they restore anoikis sensitivity, suppress anchorage‐independent survival, or target molecular dependencies of detached and metastatic GC cells.

### 4.1 Direct Modulation of Anoikis Execution and Detachment‐Induced Death

The most anoikis‐specific therapeutic concept is to restore the execution of detachment‐induced apoptosis. In GC models, caspase‐8 is a key execution node because enhancement of caspase‐8‐mediated anoikis suppresses peritoneal dissemination of human GC cells, supporting the principle that reactivation of apoptotic execution after detachment can limit serosal spread [6]. Although direct caspase‐8 restoration is not yet clinically established in GC, this finding provides a mechanistic benchmark for evaluating candidate therapies: agents should be prioritized when they induce cell death specifically under detachment or suspension conditions, increase caspase‐8/caspase‐9/caspase‐3 activation after anchorage loss, or reverse the survival advantage of anoikis‐resistant GC cells.

Several preclinical agents fit this direct anoikis‐modulating category. Celecoxib induces anoikis‐like apoptosis and suppresses Helicobacter pylori‐induced proliferation and invasion of GC cells through an adenine nucleotide translocator‐dependent mechanism, although its clinical relevance as an anoikis‐directed therapy remains exploratory [58]. Platycodin D induces anoikis and caspase‐mediated apoptosis in AGS GC cells through p38 mitogen‐activated protein kinase (MAPK) activation, representing another preclinical example of detachment‐associated apoptotic induction [59]. Emodin sensitizes GC cells to anoikis by generating reactive oxygen species and inhibiting RhoA activation, thereby linking oxidative stress, focal‐adhesion/cytoskeletal disruption, and detachment‐induced death [60]. Pseudomonas fluorescens lectin (PFL) provides a more adhesion‐focused mechanism by downregulating cell‐surface integrins and EGFR, inducing autophagy, and enhancing caspase‐8‐dependent apoptosis in GC cells [61, 62]. In addition, fatty acid synthase (FASN) knockdown suppresses anoikis resistance of GC cells through inhibition of the p‐ERK1/2/Bcl‐xL pathway, thereby connecting lipid metabolism, ERK survival signaling, and mitochondrial apoptotic priming [63].

Autophagy modulation is another direct therapeutic entry point because detached GC cells can use protective autophagy to survive suspension stress. Recent work showed that GDF15 enhances anoikis resistance and metastasis of GC through protective autophagy [37]. Consistently, circular RNA (circRNA) derived from SPECC1 (circSPECC1) promotes liquid–liquid phase separation of autophagy‐related 4B cysteine peptidase (ATG4B) and enhances ATG4B degradation, while the Food and Drug Administration (FDA)‐approved human immunodeficiency virus (HIV) protease inhibitor lopinavir enhances anoikis by remodeling autophagy in a circRNA ‐dependent manner [64]. These findings suggest that autophagy‐targeting strategies may be particularly relevant in GC cells that survive in ascites or other anchorage‐independent microenvironments. However, because autophagy can have context‐dependent tumor‐suppressive or tumor‐promoting roles, clinical translation will require biomarkers of protective autophagy, detachment‐specific assays, and careful evaluation of combination schedules. To distinguish anoikis‐focused therapeutic strategies from broadly cytotoxic or general anticancer approaches, Table 2 summarizes compounds and interventions that have been reported to induce anoikis‐like death, restore detachment‐induced apoptosis, or suppress anoikis resistance in GC, together with their target type, mechanism, experimental model, functional effect, clinical stage, and supporting references.

**Table 2:** The modulation of anoikis in GC as a therapeutic strategy.

Compound	Target type	Mechanism	Type of experiment	Function	Clinical stage	Ref.
Celecoxib	COX‐2 inhibitor (NSAID)	COX‐2 inhibitor	In vitro AGS cell line	Causes anoikis‐like death	Exploratory clinical study in GC (combination chemotherapy); no GC approval	[58]
PD	Triterpenoid saponin (natural product)	Extraction from the roots of Platycodon grandiflorus	In vitro AGS cell line	Suppresses cell proliferation and triggers anoikis	Preclinical	[59]
Lopinavir	HIV protease inhibitor (repurposed)	Antiretroviral of the protease inhibitor class	In vitro GC cell line	Augmens anoikis and inhibits metastases in a circRNA‐dependent manner	Preclinical in GC; approved for HIV (no GC trials)	[64]
Emodin	Anthraquinone derivative (natural product)	A derivative of anthraquinone	In vitroSGC‐7901 cell line	Results in inactivation of RhoA and leads to anoikis	Preclinical	[60]
RFE	Plant extract (multi‐target)	Extraction from deciduous shrub	In vitro AGS cell line	Decreases anoikis resistance via the downregulation of proteins like EGFR	Preclinical	[65]
Neratinib	Irreversible pan‐HER TKI	HER inhibitor	In vitro GC cell line	Decreases anoikis resistance via EMT markers	Phase I in GC (combination with trastuzumab deruxtecan; NCT05274048)	[66]
PFL	High‐mannose‐binding lectin (protein)	An anti‐HIV lectin	In vitro MKN28 cell line	Induces a form of cell death similar to anoikis via downregulation of integrin and EGFR	Preclinical (in vitro ± in vivo); no clinical development	[61]
TPA	PKC activator (phorbol ester)	PKC activator	In vitro MKN45 and MKN74 cell line	Induces anoikis via PKC alpha activity	Preclinical; not clinically applicable in oncology	[48]
siFASN	siRNA against FASN	An Small interfering RNA	In vitro MNK‐45 and AGS cell lines	Suppresses the anoikis resistance	Preclinical	[63]

Abbreviations: COX‐2, Cyclooxygenase‐2; EMT, epithelial‐mesenchymal transition; EGFR, epidermal growth factor receptor; GC, gastric cancer; HER, human epidermal growth factor receptor; PD, platycodin D; PFL, Pseudomonas fluorescens lectin; PKC, protein kinase C; RFE, Ribes fasciculatum; siFASN, small interfering RNA directed against Fatty acid synthase; TPA, 12‐O‐tetradecanoyl phorbol‐13‐acetate.

### 4.2 Targeting Anoikis‐Resistance Hubs Rather Than General Anticancer Pathways

Many clinically relevant targets in GC are not specific to anoikis but become therapeutically relevant when they sustain survival after detachment. Integrin/FAK/Src signaling represents the most proximal adhesion‐related targetable axis. Because FAK transduces integrin‐dependent survival signals to PI3K–AKT and MEK‐ERK pathways, FAK inhibition is mechanistically aligned with anoikis restoration. In diffuse‐type GC, preclinical data support combined FAK and MAPK pathway inhibition, and a phase II trial is evaluating defactinib plus avutometinib in advanced diffuse GC (NCT06487221) [67]. This strategy is especially relevant to anoikis biology because diffuse and EMT‐like GC phenotypes are characterized by altered adhesion, invasion, and metastatic dissemination.

The PI3K–AKT–mTOR pathway is another central anti‐anoikis hub. In GC, ITGBL1 promotes anoikis resistance and metastasis through the AKT/FBLN2 axis, and high ITGBL1 expression is associated with unfavorable prognosis [40]. Nevertheless, broad mTOR inhibition has not been successful in unselected GC populations: the phase III GRANITE‐1 trial showed that everolimus did not significantly improve overall survival in previously treated advanced GC [68]. Therefore, PI3K–AKT–mTOR targeting should not be presented as a general GC therapy but as a biomarker‐dependent strategy for tumors with pathway activation, ITGBL1/ITGB1‐high features, or detachment‐survival signatures.

YAP/TEAD signaling is particularly important because it connects mechanotransduction, cytoskeletal remodeling, anchorage‐independent survival, and peritoneal dissemination. YAP1 is essential for gastric adenocarcinoma peritoneal metastasis, and YAP1 inhibition attenuates peritoneal colonization in preclinical models [21]. More recently, GRP94 was shown to promote anoikis resistance and peritoneal metastasis through the YAP/TEAD1 pathway in GC, providing a direct mechanistic bridge between anoikis resistance and YAP/TEAD therapeutic targeting [22]. Clinically, TEAD inhibitors are entering early‐phase trials in solid tumors; VT3989, a first‐in‐class oral TEAD palmitoylation inhibitor, has shown early clinical proof‐of‐concept in refractory solid tumors, mainly mesothelioma [69]. Although not yet validated specifically in GC, TEAD inhibition is a rational strategy for YAP‐high, GRP94‐high, diffuse‐type, or peritoneal‐metastatic GC.

The BDNF/TrkB axis should also be discussed as an anoikis‐resistance pathway rather than simply a growth‐factor pathway. BDNF/TrkB promotes anoikis resistance, lymph node metastasis, peritoneal dissemination, and poor prognosis in GC [25]. Recent evidence further shows that TRIM69 suppresses anoikis resistance and metastasis through ubiquitin–proteasome‐mediated degradation of PRKCD and downstream inhibition of BDNF production [41]. These findings suggest that TrkB/BDNF‐high tumors may represent a biologically defined subgroup. However, approved TRK inhibitors such as larotrectinib and entrectinib are clinically effective primarily in tumors harboring neurotrophic receptor tyrosine kinase (NTRK) fusions, which are rare in GC. Thus, Trk pathway targeting should be based on molecular evidence of actionable NTRK fusion rather than BDNF/TrkB expression alone [70, 71].

### 4.3 Prognostic Biomarkers and Patient Stratification Based on Anoikis‐Related Pathways

The clinical value of anoikis biology in GC depends on its ability to guide prognosis and patient selection. Several anoikis‐related mediators have prognostic or stratification potential. ITGB1 is associated with poor prognosis, Wnt/β‐catenin‐related signaling, and immune suppression in GC, suggesting that ITGB1‐high tumors may represent an adhesion‐survival and immune‐excluded subgroup suitable for integrin/FAK‐centered combinations [72]. ITGBL1 is associated with EMT, AKT signaling, anoikis resistance, metastasis, and poor outcome, supporting its use as a candidate biomarker for AKT/FAK pathway dependency [40]. To strengthen the translational link between anoikis biology and clinical decision‐making, Table 3 summarizes available cohort‐based evidence for anoikis‐resistance markers in GC, highlighting their prognostic value, association with metastatic or peritoneal dissemination phenotypes, and potential utility for biomarker‐driven patient stratification.

**Table 3:** Clinical cohort analyses of anoikis resistance markers in gastric cancer.

Marker/axis	Dataset/cohort	Assay/method	Endpoint	Association	Notes	Ref.
ITGBL1 (→ AKT/FBLN2)	TCGA‐STAD; external GEO sets	Bulk RNA; KM/COX (public portals: GEPIA/UALCAN/KMplot)	OS/DFS	High ITGBL1 → worse survival	Also correlates with EMT and immune infiltration	[40]
ITGB1 (β1‐integrin)	TCGA‐STAD; ACRG/GSE62254; GSE15459	RNA; immune deconvolution	OS; immune context	High ITGB1 → poor prognosis; immune‐suppressive TME	Integrin–TME link supports ICI combinations	[72]
GRP94 → YAP/TEAD1	Hospital GC TMA + validation	IHC; transcriptomics	OS; peritoneal mets	High GRP94/YAP → worse outcomes	Mechanistic link to anoikis resistance	[22]
YAP1/TEAD activity	Peritoneal metastasis cohorts; diffuse/poorly‐cohesive GC	IHC/RNA; functional scoring	OS; peritoneal dissemination	YAP activation → poor prognosis & PC	Therapeutically actionable (TEAD inhibitors)	[21]
BDNF/TrkB (NTRK2)	Hospital GC cohort	IHC; survival analysis	OS	BDNF/TrkB co‐expression → worse OS	In vivo K252a blocks peritoneal dissemination	[25]
MYH9 → β‐catenin	Primary GC vs peritoneal metastasis	Proteomics + RNA + IHC; survival	OS; metastasis	High MYH9 → metastasis & poor outcome	Nuclear MYH9 drives CTNNB1	[36]
CAV1 (tumor vs stroma)	Multi‐cancer meta + GC subsets	IHC/meta‐analysis	OS	Tumor CAV1: mixed; Stromal CAV1↑ → longer OS	Context‐dependent; EMT/chemo‐resistance link	[54]
Anoikis‐related gene signatures (ARG)	TCGA‐STAD; GSE84437/15459/66229	LASSO signatures	OS; immune/therapy response	High ARG‐risk → worse OS; distinct immune states	Validated across external GEO cohorts	[73]

Abbreviations: ACRG, Asian Cancer Research Group; AKT, Protein kinase B (PKB); ARG, Anoikis‐Related Genes; BDNF, Brain‐Derived Neurotrophic Factor; CAV1, Caveolin‐1; CTNNB1, Catenin Beta‐1 (β‐Catenin); DFS, Disease‐Free Survival; EMT, Epithelial–Mesenchymal Transition; FBLN2, Fibulin‐2; GC, Gastric Cancer; GEO, Gene Expression Omnibus; GRP94, Glucose‐Regulated Protein 94 (HSP90B1; endoplasmin/gp96); HIF‐1α, Hypoxia‐Inducible Factor‐1 alpha (HIF1A); ICI, Immune Checkpoint Inhibitor; IHC, Immunohistochemistry; ITGA5, Integrin Alpha‐5 (CD49e); ITGB1, Integrin Beta‐1 (CD29); ITGBL1, Integrin Beta‐Like 1 (TIED); KM, Kaplan–Meier; KMplot, Kaplan–Meier Plotter database; LASSO, Least Absolute Shrinkage and Selection Operator; MEK, MAPK/ERK Kinase (MAP2K1/2); MYH9, Myosin Heavy Chain 9 (non‐muscle myosin IIA heavy chain); NF‐κB, Nuclear Factor kappa‐B; NTRK2, Neurotrophic Receptor Tyrosine Kinase 2 (TrkB); OS, Overall Survival; RNA, Ribonucleic Acid; SP1, Specificity Protein 1; STAD, Stomach Adenocarcinoma; TCGA, The Cancer Genome Atlas; TEAD1, TEA Domain Transcription Factor 1; TMA, Tissue Microarray; TME, Tumor Microenvironment; TrkB, Tropomyosin Receptor Kinase B; UALCAN, University of Alabama Cancer Data Portal; YAP, Yes‐Associated Protein 1 (YAP1).

GRP94 and YAP/TEAD1 activity are linked to peritoneal metastasis and unfavorable prognosis, suggesting that GRP94‐high or YAP‐active GC may be suitable for TEAD/YAP‐directed therapeutic exploration [22]. BDNF/TrkB expression is associated with systemic dissemination and adverse clinical features, although its therapeutic implication requires distinction between ligand–receptor overexpression and true NTRK fusion dependency [25].

Beyond single markers, anoikis‐related gene signatures may help stratify GC patients. Recent transcriptomic studies have developed anoikis‐related gene signatures that predict prognosis, immune microenvironment features, and potential therapeutic response in GC [73]. These signatures should be integrated with established molecular classifications such as The Cancer Genome Atlas (TCGA) and Asian Cancer Research Group (ACRG) subtypes. For example, EMT/microsatellite stable (MSS) or diffuse‐type tumors may be enriched for adhesion remodeling, FAK activation, immune exclusion, and peritoneal dissemination, whereas YAP/TEAD‐high tumors may show stronger mechanotransduction and anchorage‐independent survival programs. EMT‐related signatures can also predict prognosis and response to immunotherapy in GC, supporting the use of EMT/anoikis combined stratification in future trials [74].

### 4.4 Clinical Translation and Trial‐Oriented Therapeutic Strategies

Clinical translation should prioritize pathway‐defined strategies rather than unselected use of anti‐cancer agents. First, FAK‐centered combinations should be evaluated in diffuse‐type, EMT‐high, ITGB1/ITGBL1‐high, or peritoneal‐metastatic GC. Defactinib plus avutometinib is particularly relevant because it simultaneously targets FAK‐dependent adhesion survival and MAPK pathway compensation, a mechanism aligned with anoikis resistance in diffuse GC [67]. Future trials should incorporate detachment‐survival biomarkers, phospho‐FAK, phospho‐ERK, integrin signatures, and ascites‐derived tumor cell assays as correlative endpoints.

Second, YAP/TEAD‐directed therapy should be considered for YAP‐high or GRP94‐high GC, especially in peritoneal dissemination. Because YAP/TEAD signaling supports anchorage‐independent survival, stemness, and peritoneal colonization, TEAD inhibitors should be tested with pharmacodynamic readouts such as connective tissue growth factor (CTGF)/cysteine‐rich angiogenic inducer 61 (CYR61) expression, YAP nuclear localization, TEAD target suppression, and changes in ascites tumor‐cell viability. Combination with fluoropyrimidines, FAK inhibitors, or immune checkpoint blockade may be rational, but should be restricted to biomarker‐enriched cohorts until GC‐specific clinical evidence becomes available [21, 22, 69].

Third, PI3K–AKT–mTOR targeting should be biomarker‐guided. The failure of everolimus to improve overall survival in unselected advanced GC indicates that pathway inhibition alone is insufficient without molecular enrichment [68]. Therefore, future studies should prioritize tumors with AKT activation, phosphatidylinositol‐4,5‐bisphosphate 3‐kinase catalytic subunit alpha (PIK3CA)/AKT pathway alterations, ITGBL1‐high signatures, or anoikis‐related AKT dependence. Combination strategies with chemotherapy, FAK inhibition, or autophagy modulation may be more appropriate than single‐agent mTOR inhibition.

Fourth, autophagy‐modulating approaches should be developed for tumors with detachment‐induced protective autophagy. The GDF15/autophagy and circSPECC1/ATG4B/lopinavir findings suggest that anoikis‐resistant GC cells may depend on autophagy during suspension survival [37, 64]. These strategies may be especially relevant for malignant ascites and peritoneal dissemination, where tumor cells experience anchorage loss, nutrient stress, and oxidative stress. However, clinical translation requires careful patient selection and functional validation because autophagy inhibition may have context‐dependent effects.

Finally, HER/EGFR‐directed therapy may intersect with anoikis resistance when EGFR–integrin cooperation sustains survival after detachment. Neratinib reduces invasion, EMT features, and anoikis resistance in gastric adenocarcinoma cells [66], and a phase I trial is evaluating neratinib plus trastuzumab deruxtecan in advanced refractory gastric and esophageal cancer with human epidermal growth factor receptor/human epidermal growth factor receptor 2 (HER2) expression or alteration. This approach should be discussed as indirect targeting of anoikis‐resistance signaling rather than as a therapy designed specifically to induce anoikis. These pathway‐specific options are summarized in Table 4, which is intended to function as a translational bridge between the mechanistic evidence reviewed above and biomarker‐defined treatment selection. The table distinguishes strategies that may directly restore detachment‐induced death from approaches that indirectly suppress anoikis‐resistance hubs, and links each strategy to the most plausible GC subgroup, evidence level, and remaining validation need.

**Table 4:** Therapeutic strategies targeting anoikis resistance in gastric cancer.

Therapeutic class	Anoikis relevance	Example	Evidence level	Suggested patient subgroup	Ref.
Caspase/anoikis execution restoration	Direct restoration of detachment‐induced apoptosis	Caspase‐8 activation	Preclinical GC peritoneal dissemination model	Peritoneal dissemination	[6]
Integrin/EGFR surface depletion	Direct disruption of adhesion‐survival signaling	Pseudomonas fluorescens lectin (PFL)	Preclinical GC models	EGFR/integrin‐high GC	[61, 62]
Autophagy modulation	Direct inhibition of protective suspension‐survival autophagy	Lopinavir/circSPECC1/ATG4B; GDF15/autophagy	Preclinical GC models	Ascites/peritoneal disease; autophagy‐high tumors	[37, 64]
RhoA/cytoskeletal stress modulation	Direct induction of anoikis through focal‐adhesion/cytoskeletal disruption	Emodin‐induced ROS/RhoA inhibition	Preclinical GC models	RhoA/focal‐adhesion‐dependent GC	[60]
Caspase‐mediated anoikis induction	Direct induction of anoikis‐like apoptotic death	Platycodin D; celecoxib	Preclinical GC models	Exploratory; anoikis‐resistant GC models	[58, 59]
FAK/MAPK inhibition	Indirect targeting of adhesion‐survival hubs	Defactinib + avutometinib	Phase II trial in diffuse GC; preclinical rationale	Diffuse‐type, EMT‐high, ITGB1/ITGBL1‐high GC	[67] NCT06487221
YAP/TEAD inhibition	Indirect targeting of mechanotransduction‐dependent anoikis resistance	VT3989; verteporfin as tool compound	Early‐phase solid tumor trials; preclinical GC evidence	YAP‐high, GRP94‐high, peritoneal‐metastatic GC	[21, 22, 69]
PI3K–AKT–mTOR inhibition	Indirect targeting of survival signaling downstream of integrins/ITGBL1	AKT/mTOR inhibitors; everolimus as negative unselected example	Mixed evidence; everolimus negative in unselected GC	AKT‐activated or ITGBL1‐high tumors	[40, 68]
HER/EGFR targeting	Indirect targeting of EGFR–integrin cooperation and EMT‐associated survival	Neratinib	Preclinical GC evidence; clinical relevance mainly in HER2/EGFR‐driven disease	HER2/EGFR‐driven tumors	[66]
FASN/ERK/Bcl‐xL targeting	Indirect targeting of metabolic‐survival signaling linked to anoikis resistance	FASN knockdown	Preclinical GC models	FASN‐high or ERK/Bcl‐xL‐dependent GC	[63]
Trk pathway targeting	Context‐dependent; relevant when neurotrophin signaling is actionable	Larotrectinib/entrectinib for NTRK fusion‐positive tumors	Tumor‐agnostic clinical use; NTRK fusions are rare in GC	NTRK fusion‐positive GC, not merely BDNF/TrkB overexpression	[25, 70, 71]
Biomarker‐guided anoikis‐risk stratification	Prognostic and predictive enrichment rather than direct drug targeting	ITGB1, ITGBL1, anoikis‐related gene signatures, EMT signatures	Retrospective/transcriptomic clinical evidence	High‐risk, EMT‐high, immune‐suppressed, or anoikis‐signature‐high GC	[40, 72, 73, 74]

Abbreviations: AKT, protein kinase B; ATG4B, autophagy‐related 4B cysteine peptidase; Bcl‐xL, B‐cell lymphoma‐extra large; BDNF, brain‐derived neurotrophic factor; circSPECC1, circular RNA derived from SPECC1; EGFR, epidermal growth factor receptor; EMT, epithelial–mesenchymal transition; ERK, extracellular signal‐regulated kinase; FAK, focal adhesion kinase; FASN, fatty acid synthase; GC, gastric cancer; GDF15, growth differentiation factor 15; GRP94, glucose‐regulated protein 94; HER, human epidermal growth factor receptor; ITGB1, integrin beta 1; ITGBL1, integrin beta‐like 1; MAPK, mitogen‐activated protein kinase; mTOR, mechanistic target of rapamycin; NCT, National Clinical Trial identifier; NTRK, neurotrophic receptor tyrosine kinase; PFL, Pseudomonas fluorescens lectin; PI3K, phosphoinositide 3‐kinase; RhoA, Ras homolog family member A; ROS, reactive oxygen species; TEAD, TEA domain transcription factor; Trk, tropomyosin receptor kinase; YAP, yes‐associated protein.

### 4.5 Proposed Therapeutic Framework

Based on current evidence, anoikis‐targeted strategies in GC should be biomarker‐guided. Diffuse‐type, EMT‐high, ITGB1/ITGBL1‐high, or FAK/MAPK‐activated tumors may benefit from FAK‐centered combinations. Tumors with peritoneal dissemination, nuclear YAP, GRP94 overexpression, or high TEAD target‐gene expression may be suitable for YAP/TEAD‐directed strategies. PI3K–AKT–mTOR‐based combinations should be restricted to tumors with AKT activation or ITGBL1/AKT/FBLN2 signatures, whereas GDF15‐high or ATG4B/autophagy‐dependent tumors may be candidates for autophagy‐modulating approaches. Finally, BDNF/TrkB activation should be distinguished from actionable NTRK fusions, because approved TRK inhibitors should be guided by fusion status rather than receptor expression alone.

Building on the therapeutic categories summarized in Table 4, this proposed framework clarifies that anoikis‐targeted therapy in GC is not equivalent to general apoptosis induction or broad anticancer treatment. The most relevant strategies are those that restore detachment‐induced death, disable compensatory adhesion‐survival signaling, suppress anchorage‐independent transcriptional programs, or exploit vulnerabilities of suspended and peritoneally disseminated tumor cells.

Figure 3 integrates these therapeutic concepts with the mechanistic hierarchy discussed throughout the manuscript. Specifically, the figure links ECM detachment and anoikis execution to GC‐specific resistance mediators, including integrin/FAK/Src, PI3K‐AKT, MEK‐ERK, NF‐κB, Wnt/β‐catenin, YAP/TEAD, BDNF/TrkB, and autophagy‐related pathways. In this way, Table 4 provides the treatment‐oriented summary, whereas Figure 3 provides the mechanistic map that explains why particular biomarker‐defined subgroups may be suitable for specific therapeutic strategies. Future clinical studies should therefore include anoikis‐related biomarkers, ascites‐derived tumor‐cell models, patient‐derived organoids, and detachment‐specific pharmacodynamic endpoints to determine whether candidate therapies truly modulate anoikis resistance in GC.

**Figure 3:**
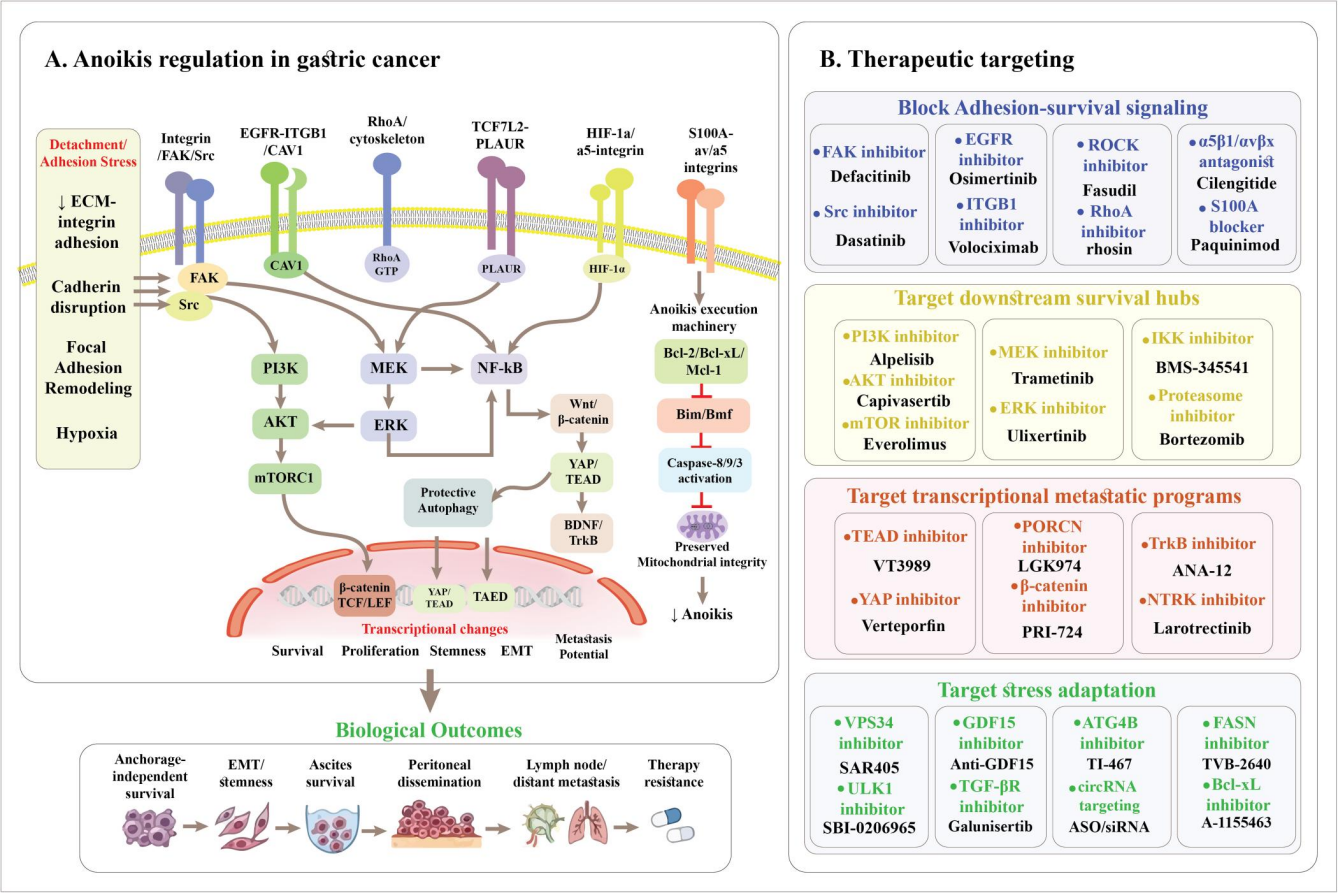
Integrated schematic model of anoikis regulation and therapeutic targeting in gastric cancer. This figure summarizes anoikis regulation and therapeutic vulnerabilities in gastric cancer (GC). (A) illustrates the regulatory network through which detached GC cells evade anoikis. Loss or alteration of ECM–integrin and cadherin‐mediated adhesion initiates focal adhesion remodeling, cytoskeletal reorganization, altered mechanotransduction, and compensatory activation of adhesion‐survival signaling. Upstream regulators, including integrin/FAK/Src, EGFR–ITGB1/CAV1, RhoA‐related cytoskeletal remodeling, TCF7L2–PLAUR, HIF‐1α/α5‐integrin, and S100A4–integrin signaling, converge on core survival hubs such as PI3K–AKT–mTOR, MEK‐ERK, NF‐κB, Wnt/β‐catenin, YAP/TEAD, BDNF/TrkB, and protective autophagy. GC‐specific mediators, including MYH9–CTNNB1, ITGBL1–AKT/FBLN2, GRP94–YAP/TEAD1, GDF15/autophagy, TRIM69/PRKCD/BDNF, and anoikis‐related miRNAs, further reinforce anchorage‐independent survival. These pathways suppress anoikis execution by maintaining mitochondrial integrity, increasing anti‐apoptotic Bcl‐2 family signaling, and limiting caspase‐8, caspase‐9, and caspase‐3 activation, thereby promoting EMT, ascites survival, peritoneal dissemination, lymph node metastasis, and therapy resistance. (B) shows therapeutic strategies onto these regulatory nodes, including restoration of detachment‐induced apoptosis, inhibition of integrin/FAK/Src and EGFR–integrin signaling, targeting of PI3K–AKT–mTOR, MEK‐ERK, YAP/TEAD, Wnt/β‐catenin, BDNF/TrkB, and autophagy‐related pathways, and biomarker‐guided patient stratification based on ITGB1/ITGBL1, GRP94/YAP–TEAD activity, EMT/diffuse‐type features, GDF15/autophagy dependence, BDNF/TrkB activation, NTRK fusion status, and anoikis‐related gene signatures. This model emphasizes that anoikis resistance in GC is a network‐level metastatic survival program and that therapeutic intervention should be matched to the dominant resistance module rather than applied as nonspecific apoptosis induction. Abbreviations: AKT, protein kinase B; BDNF, brain‐derived neurotrophic factor; CAV1, caveolin‐1; ECM, extracellular matrix; EGFR, epidermal growth factor receptor; EMT, epithelial–mesenchymal transition; ERK, extracellular signal‐regulated kinase; FAK, focal adhesion kinase; FASN, fatty acid synthase; FBLN2, fibulin‐2; GC, gastric cancer; GDF15, growth differentiation factor 15; GRP94, glucose‐regulated protein 94; ITGB1, integrin beta 1; ITGBL1, integrin beta‐like 1; mTOR, mechanistic target of rapamycin; NF‐κB, nuclear factor kappa B; NTRK, neurotrophic receptor tyrosine kinase; PI3K, phosphoinositide 3‐kinase; PRKCD, protein kinase C delta; TEAD, TEA domain transcription factor; TrkB, tropomyosin receptor kinase B; YAP, yes‐associated protein.

## 5 Conclusion and Future Direction

Anoikis escape is increasingly recognized as a central enabler of peritoneal dissemination and treatment refractoriness in GC, because detached tumor cells must survive loss of ECM attachment before colonizing the peritoneal cavity or distant metastatic niches [3, 8]. Mechanistically, this process can be understood as a sequential transition from adhesion‐loss sensing to apoptotic execution failure and compensatory survival rewiring, with integrin/FAK‐PI3K‐AKT, Wnt/β‐catenin, YAP/TEAD, and BDNF/TrkB acting as major interconnected hubs [18, 21, 25, 36]. GC‐specific studies support this network model by showing that MYH9/CTNNB1, GRP94/YAP–TEAD1, TCF7L2/PLAUR, ITGBL1/AKT/FBLN2, and TRIM69/PRKCD/BDNF can regulate anoikis resistance, metastasis, or peritoneal dissemination [22, 26, 36, 40, 41]. Because these pathways function as a network rather than as isolated nodes, durable therapeutic control is unlikely to be achieved through single‐agent blockade, and biomarker‐guided combinations should therefore be prioritized.

Three near‐term translational avenues are proposed on mechanistic grounds. First, YAP/TEAD‐directed strategies should be prioritized in YAP‐high tumors and peritoneal disease, because YAP1 promotes gastric adenocarcinoma peritoneal metastasis and YAP1 inhibition attenuates peritoneal tumor growth in preclinical models [21]. This rationale is further supported by the GRP94/YAP–TEAD1 axis, in which GRP94 promotes anoikis resistance and peritoneal metastasis through YAP/TEAD1 signaling in GC [22]. TEAD inhibition is an emerging therapeutic strategy for YAP/TAZ‐driven cancers, although compensatory PI3K‐AKT activation may limit TEAD inhibitor efficacy and supports rational combination approaches rather than TEAD blockade alone [75, 76]. Second, FAK‐centered combinations should be tested in mesenchymal, MSS/EMT‐like, ITGB1/ITGBL1‐high, or peritoneal‐metastatic GC, because FAK inhibition can reduce stromal barriers and improve immune‐cell infiltration in immunotherapy‐resistant solid tumor models [77]. In GC, this strategy should be integrated cautiously with current programmed cell death protein 1 (PD‐1)‐based treatment backbones, as pembrolizumab plus chemotherapy has improved outcomes in advanced HER2‐negative gastric or gastroesophageal junction cancer, but FAK plus checkpoint blockade still requires GC‐specific validation [78]. Third, PI3K‐AKT‐mTOR‐based regimens should be confined to biomarker‐enriched cohorts, because ITGBL1 promotes GC anoikis resistance through the AKT/FBLN2 axis, whereas unselected mTOR inhibition with everolimus failed to improve overall survival in the phase III GRANITE‐1 trial [40, 68].

Mechanistic crosstalk between anoikis resistance, autophagy, and ferroptosis provides an additional basis for rational combinations. Autophagy can protect detached GC cells from anoikis, and the ELAV‐like RNA‐binding protein 1 (ELAVL1)/circSPECC1/ATG4B axis has been shown to mediate autophagy‐dependent anoikis resistance, with lopinavir enhancing anoikis and suppressing metastasis by remodeling this pathway [64]. GDF15 has also been reported to promote GC anoikis resistance and metastasis through protective autophagy, supporting further evaluation of autophagy‐modulating approaches in detachment‐stressed and ascites‐derived tumor cells [37]. Ferroptosis‐oriented strategies may be relevant because glutathione peroxidase 4 (GPX4) regulates ferroptosis and has prognostic and functional significance in GC, while ferroptosis pathways are increasingly linked to gastrointestinal cancer progression and treatment resistance [79, 80]. Future studies should therefore test whether YAP/FAK blockade, autophagy modulation, and ferroptosis induction produce true synergy in patient‐derived organoids, ascites‐adapted cultures, and peritoneal metastasis models rather than relying only on standard adherent cell culture systems.

Critical knowledge gaps persist at the interface of epigenetic regulation, non‐coding RNAs, and immune integration of the detachment‐survival phenotype. miR‐204 suppresses SIRT1‐driven EMT, invasion, and anoikis resistance in GC cells, while miR‐335‐5p and miR‐497 act as metastasis‐ or anoikis‐suppressive miRNAs through PLAUR/CDH11‐ and Wnt/β‐catenin‐related mechanisms [50, 52, 81]. Genome‐scale clustered regularly interspaced short palindromic repeats (CRISPR) interference/activation screens may help identify upstream chromatin regulators, non‐coding RNAs, and immune‐modulatory dependencies that determine whether GC cells remain anoikis‐sensitive or acquire detachment survival [82]. Epigenetic or dual‐targeting approaches should then be evaluated only after such dependency mapping; for example, dual FAK‐histone deacetylase (HDAC) inhibition has shown preclinical activity in GC, but its role in restoring detachment sensitivity remains to be tested directly [83]. Within the neurotrophin axis, BDNF/TrkB overexpression should also be distinguished from actionable NTRK fusion dependency, because approved TRK inhibitors are most strongly supported in NTRK fusion‐positive cancers rather than in tumors with receptor overexpression alone [25, 84].

Methodological rigor will be essential for reproducibility and translation. Future studies should routinely use authenticated GC cell lines, patient‐derived organoids, ascites‐derived tumor cells, and in vivo systems that recapitulate serosal spread, rather than relying only on legacy suspension assays or poorly authenticated models. Clinically, composite biomarkers integrating YAP/TEAD activity, integrin/FAK signaling, ITGBL1/AKT dependence, miRNA panels, autophagy markers, and circulating or ascites‐derived tumor‐cell readouts should be developed to guide patient selection. Endpoints should also reflect serosal disease biology, including peritoneal carcinomatosis burden, ascites control, detachment‐specific pharmacodynamic changes, and patient‐reported symptom burden, rather than Response Evaluation Criteria in Solid Tumors (RECIST)‐based tumor shrinkage alone. Through these mechanism‐anchored proposals, biomarker‐guided multimodal regimens may be advanced to disable detachment survival while reprogramming the tumor–stroma–immune ecosystem in GC.

## Author Contributions

**Jian Zhong:** writing – review and editing. **Xing Qi:** conceptualization, writing – review and editing, writing – original draft. **Honglei Wu:** investigation, validation, writing – review and editing, supervision. **Zheng Lei:** visualization, writing – review and editing, supervision.

## Funding

The authors have nothing to report.

## Conflict of Interests

The authors declare no conflicts of interest.

## AI Using

No paper mill and no artificial intelligence were used.

## Data Availability

Data sharing not applicable to this article as no datasets were generated or analyzed during the current study.
